# New Insights into the Role of Inflammation in the Pathogenesis of Atherosclerosis

**DOI:** 10.3390/ijms18102034

**Published:** 2017-09-22

**Authors:** Meng-Yu Wu, Chia-Jung Li, Ming-Feng Hou, Pei-Yi Chu

**Affiliations:** 1 Department of Emergency Medicine, Taipei Tzu Chi Hospital, Buddhist Tzu Chi Medical Foundation, New Taipei 231, Taiwan; skyshangrila@gmail.com; 2 Department of Emergency Medicine, School of Medicine, Tzu Chi University, Hualien 970, Taiwan; 3 Research Assistant Center, Show Chwan Memorial Hospital, Changhua 500, Taiwan; nigel6761@gmail.com; 4 Department of Surgery, College of Medicine, Kaohsiung Medical University, Kaohsiung 807, Taiwan; 5 Department of Surgery, Kaohsiung Municipal Hsiao Kang Hospital, Kaohsiung 807, Taiwan; 6 Division of Breast Surgery, Kaohsiung Medical University Hospital, Kaohsiung 807, Taiwan; 7 Department of Pathology, Show Chwan Memorial Hospital, Changhua 500, Taiwan; 8 School of Medicine, College of Medicine, Fu-Jen Catholic University, New Taipei 242, Taiwan; 9 National Institute of Cancer Research, National Health Research Institutes, Tainan 704, Taiwan

**Keywords:** atherosclerosis, atherosclerotic immunity, atherosclerotic microenvironment, oxidative stress, macrophage

## Abstract

Atherosclerosis is a chronic inflammatory disease characterized by the accumulation of lipids, smooth muscle cell proliferation, cell apoptosis, necrosis, fibrosis, and local inflammation. Immune and inflammatory responses have significant effects on every phase of atherosclerosis, and increasing evidence shows that immunity plays a more important role in atherosclerosis by tightly regulating its progression. Therefore, understanding the relationship between immune responses and the atherosclerotic microenvironment is extremely important. This article reviews existing knowledge regarding the pathogenesis of immune responses in the atherosclerotic microenvironment, and the immune mechanisms involved in atherosclerosis formation and activation.

## 1. Introduction

Atherosclerosis is a chronic inflammatory disease characterized by intense immunological activity. Atherosclerosis is widespread, and leads to morbidity and mortality along with the development of circulatory problems, including coronary artery disease and cerebrovascular disease [1]. In the heart, atherosclerosis can cause myocardial infarction and heart failure due to coronary artery stenosis, and in the brain, the stenosis or rupture of atherosclerotic plaques leads to transient ischemic attacks, ischemic stroke, or hemorrhage stroke. If stenosis affects renal arterial branches, it can result in renal impairment and general hypertension. On other arterial branches of extremities, atherosclerosis can lead to peripheral arterial occlusion disease and critical limb ischemia. The formation of atherosclerotic plaques, which are characterized by the accumulation of lipids, local inflammation, smooth muscle cell (SMC) proliferation, cell apoptosis, necrosis, and fibrosis, is a major causative factor of arterial stenosis, and involves a chronic inflammatory response initiated by endothelial damage and inflammatory cell activation [2]. This review article summarizes current knowledge regarding the role of immunity in atherosclerosis and, in particular, the pathogenesis of human atherosclerosis. The atherosclerotic microenvironment and mechanisms associated with immune responses to atherosclerosis are also discussed in detail.

## 2. Lipid Metabolism and Low-Density Lipoprotein (LDL) Modification

### 2.1. Roles of Lipid Metabolism in Atherosclerosis

Cholesterol, triglycerides, and lipoproteins are implicated in atherosclerosis pathogenesis. Increased serum low-density lipoprotein (LDL) and triglyceride concentrations are responsible for the formation of atherosclerotic lesions [3]. The roles of lipid metabolism and LDL modification are important for atherosclerosis development. Lipid metabolism occurs via exogenous and endogenous pathways. The exogenous pathway begins with chylomicron synthesis and secretion by the intestine. Chylomicrons contain apolipoproteins (Apos) B-48, C-II, and E. The Apo C-II is an essential cofactor of lipoprotein lipase (LPL) transport of fatty acids to adipose tissue. After LPL activity, the chylomicron remnant is relatively enriched in cholesterol due to the loss of triacylglycerol, and absorbed into the liver by Apo E. The endogenous pathway begins with the synthesis of very LDL (VLDL) particles, which are triglyceride-rich and contain Apo B-100, C-II, and E. After removal of the triglycerides to adipose tissue, a portion of VLDL remnants are metabolized to LDL particles [4] that transport cholesterol esters and lesser amounts of triglycerides. Small LDL particles penetrate the endothelial barrier and deposit in the extracellular matrix of subendothelial spaces by Apo B-100 binding to proteoglycans, which are among the most important molecules for lipoprotein retention [5,6,7]. Retention of LDL particles in the vessel wall is considered the first step in atherosclerosis pathogenesis [8]. 

### 2.2. LDL Modification and Oxidization in Atherosclerosis

In the second step of atherosclerosis pathogenesis, subendothelial LDL is oxidized by resident vascular cells, and triggers vascular-cell production of monocyte chemoattractant protein-1 (MCP-1) and macrophage colony stimulating factors (M-CSFs) [9]. Under smoking, hypertension, hyperglycemia, and hyperlipidemia status, the production of reactive oxygen species (ROS) is increased, and overwhelms the endogenous antioxidant response. The oxidative stress increases LDL oxidation and impairs endothelial function [10,11,12]. Chronic oxidative stress is a stronger risk of atherosclerosis formation. In the initial phase of LDL modification, the lipid components are interacted with ROS, and produce many types of lipid oxidation products [13]. The lipid oxidation products, such lysophospholipid products, attack with the Apo B protein. Apo B are oxidized, and lead to changes in the amino acid side chains by breaking peptide bonds. After modification of Apo B, oxidized LDL (ox-LDL) becomes a ligand of scavenger receptors [14]. The retention of ox-LDL is recognized, and interacts with two major scavenger receptors on macrophages: class A scavenger receptors (SR) AI/II, and class B scavenger receptor cluster of differentiation 36 (CD36). The SRAI/II recognizes the modification of Apo B protein on ox-LDL, and oxidized phospholipids are recognized by CD36. After interaction with scavenger receptors, the macrophages are activated and uptake ox-LDL.

**Figure 1:**
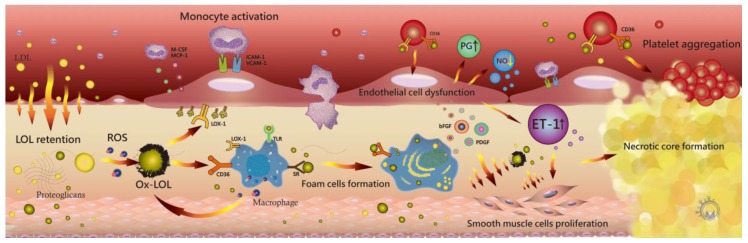
Small low-density lipoproteins (LDLs) penetrate the endothelial barrier and bind to proteoglycans via apolipoprotein B100 to retain in the subendothelial space. LDL is oxidized (ox-LDL) and induces several pro-inflammatory conditions via lectin-like oxidized LDL receptor-1 (LOX-1). The upregulation of intercellular adhesion molecule-1 (ICAM-1) and vascular-cell adhesion molecule-1 (VCAM-1) by ox-LDL increase monocyte and inflammatory cell adhesion on the endothelium. Ox-LDL particles stimulate endothelial cells and smooth muscle cells (SMCs) to secrete monocyte chemotactic protein-1 (MCP-1) and monocyte colony stimulating factor (M-CSF), with both factors inducing monocyte recruitment. Ox-LDL promotes an increased in reactive oxygen species (ROS) and inhibits nitric oxide production. Monocytes differentiate into macrophages and express scavenger receptors (SRs), cluster of differentiation 36 (CD36), LOX-1, and Toll-like receptors (TLRs). Ox-LDL–CD36 interaction induces monocyte differentiation, macrophage activation, and macrophage retention, and macrophage SRs increase ox-LDL uptake and foam-cell formation. The retention of ox-LDL leads to foam cell apoptosis and inflammatory progression. Ox-LDLs also increase the expression of growth factors, including platelet-derived growth factor (PDGF) for migration and basic fibroblast growth factor (bFGF) for proliferation, on SMCs. SMC proliferation contributes to the thickening of atherosclerotic plaques and formation of a necrotic core. The ox-LDL–CD36 interaction in resting platelets causes platelet aggregation and activation, with activated platelets expressing LOX-1 to mediate adhesion to endothelial cells and enhance endothelin-1 release. The endothelial function is impaired along with decreasing nitric oxide production and increasing prostaglandin synthesis.

Ox-LDL also induces several pro-inflammatory conditions via a lectin-like oxidized LDL receptor-1 (LOX-1) [15]. The chemotactic activity from the oxidative modification of LDL stimulates monocyte binding to endothelial cells, thereby increasing the adhesive properties of the endothelium, including the overexpression of intercellular adhesion molecule-1 (ICAM-1) and vascular cell adhesion molecule-1 (VCAM-1) [16,17,18]. Inflammatory cells and monocytes subsequently release MCP-1 to activate leukocytes in the media and stimulate the proliferation of SMCs. The monocytes differentiate into macrophages with the expression of scavenger receptors (SRs), including SR-AI/II, SR-BI, CD36, LOX-1, and Toll-like receptors (TLRs), and lead to lipid accumulation [19]. Ox-LDL–CD36 interaction induces increased ox-LDL internalization and activates additional macrophages (Figure 1). Recently reported data support ox-LDL participation in not only macrophage activation, but also retention [20,21,22,23,24]. Moreover, macrophages induce inflammation progression via interleukin (IL)-1β, tumor necrosis factor (TNF), reactive oxygen species (ROS), and metalloproteases [25,26].

Ox-LDLs also increase the expression of growth factors, including platelet-derived growth factor for migration and basic fibroblast growth factor, for proliferation on SMCs [27,28,29]. SMC proliferation contributes to the thickening of atherosclerotic plaques and the formation of a necrotic core [30]. Activation of inflammatory cytokines and ox-LDL influence extracellular matrix remodeling and cause lesion thinning by increasing matrix metalloproteinase (MMP)-1 and -9 expression. During LDL oxidation, β-cleavage reactions modulated by ROS lead to the formation of aldehydic end products, which are considered important toxic messengers and cause oxidative stress injury in vessel walls. The stress response induces SMC apoptosis via p38 mitogen-activated protein kinase and c-Jun N-terminal kinase [31,32,33]. Finally, ox-LDL–CD36 interaction also induces P-selectin expression and the activation of integrin αIIbβ3 in resting platelets, causing platelet aggregation and activation via Src kinases and Rho kinase signaling pathways [30,34,35]. The activated platelets express LOX-1 to mediate adhesion to endothelial cells [36,37], and LOX-1 expression enhances endothelin-1 (ET-1) release [38,39]. Endothelial function is impaired along with decreased nitric oxide (NO) production and increased prostaglandin synthesis [40,41]. According to previous reports, ox-LDL also plays a key role in atherosclerosis progression [1,3,22,23,24,30,33,35].

## 3. Endothelial Function and Microenvironment

Vascular endothelium plays a key role in the regulation of vascular tone [42], as well as regulation of vascular SMC (VSMC) proliferation, vascular fibrinolysis, and adhesion and migration of inflammatory cells by secreting numerous substances, including NO, ET-1, prostaglandin, endothelium-derived hyperpolarizing factor, thromboxane A_2_, and angiotensin II. Endothelial dysfunction is a primary step in atherosclerosis development. In early stage atherosclerosis, oxidative stress can originate from hyperlipidemic states, diabetes mellitus, smoking, or hypertension due to the accumulation of advanced glycation end products (AGEs) [43], which causes endothelial damage by activating cytokines and increasing LDL retention via a macrophage–SR pathway. A dysfunctional endothelium also accelerates ROS generation and induces local inflammation [44]. The dysfunction of vascular–tone balance is noted by increased ET-1 and decreased NO [45,46]. A series of pathophysiologic changes and mechanism are discussed later.

### 3.1. Roles of NO in Atherosclerosis

**Figure 2:**
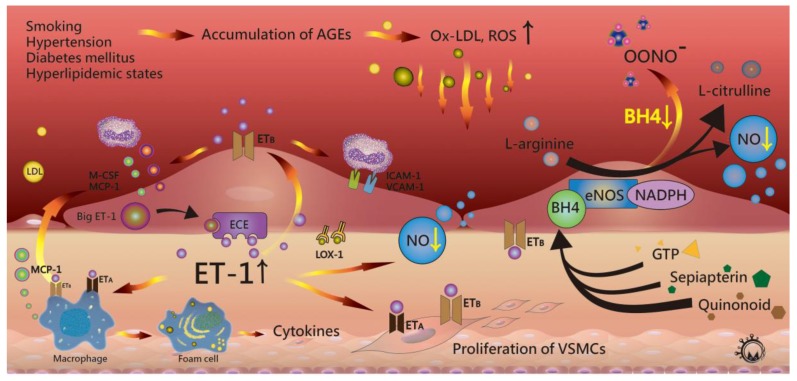
Hyperlipidemic status and other conditions can induce the accumulation of advanced glycation end products (AGEs), leading to increase reactive oxygen species (ROS) and retention of oxidized low-density lipoprotein (ox-LDL). Oxidative stress causes endothelial dysfunction and impairs the release of nitric oxide (NO) and endothelin-1 (ET-1). In atherosclerotic lesions, elevated tissue levels of ET-1 bind to ET_B_ receptors on endothelial cells and cause expression of endothelial cell adhesion molecules, such as intercellular adhesion molecule-1 (ICAM-1) and vascular-cell-adhesion molecule-1 (VCAM-1). ET-1 promotes monocyte migration and activation by monocyte chemoattractant protein-1 (MCP-1), which is released from activated macrophages and endothelial cells. ET-1 also activates vascular smooth muscle cells (VSMCs) via ET_A_ receptors to promote SMC proliferation. Oxidative stress also causes lower tissue levels of BH4 and induces the uncoupling of endothelial nitric oxide synthase (eNOS) and superoxide.

NO is a potent oxidant that regulates vascular tone, blood-pressure homeostasis, fibrinolysis, and resting-platelet and leukocyte adhesion. NO also plays a protective role by inhibiting the abnormal proliferation of VSMCs [47,48]. NO is produced from the conversion of l-arginine from endothelial cells to l-citrulline. The activity of the enzyme, nicotinamide adenine dinucleotide phosphate (NADPH)-dependent NO synthase (NOS), is mediated by calcium, flavin adenine dinucleotide, flavin mononucleotide, and tetrahydrobiopterin (BH4) as cofactors [49,50,51,52]. Current studies support two pathways for the endothelial isoform of NOS (eNOS) in atherogenesis [53]. Under physiological conditions, BH4, an antiatherogenic molecule in tissue, regulates eNOS activity to activate NO production. However, under hypertension, hypercholesterolemia, smoking, and diabetes mellitus conditions, enhance oxidative stress oxidizes BH4, leading to BH4 deficiency. Lower BH4 levels in tissue induces the uncoupling of NOS and superoxide, causing endothelial-cell damage [54] (Figure 2).

In endothelial cells, BH4 concentration plays an important role in NO synthesis. BH4 is synthesized from three pathways that regulate its production and degradation. First, BH4 is produced from guanosine 5′-triphosphate (GTP) by GTP cyclohydrolase I. Second, BH4 can be synthesized from quinonoid dihydrobiopterin (BH2), which is the natural product from the oxidation of BH4, through a salvage pathway [55]. Third, BH4 can be also synthesized from sepiapterin, which is an oxidized BH4 analogue, by enzymatic reduction of sepiapterin reductase and dihydrofolate reductase [53]. During pro-atherosclerosis conditions, the activity of GTP cyclohydrolase I decreases through ox-LDL accumulation, and BH4 bioavailability can be rapidly reduced by ROS and oxidized via the BH3 radical to BH2 [56]. Additionally, Kruppel-like factor 2 is inhibited by inflammatory cytokines and causes expression of VCAM-1, the endothelial adhesion molecule, and promotes laminar shear stress in endothelial cells [57].

### 3.2. Role of ET-1 in Atherosclerosis

ET-1 is a 21-amino acid peptide that regulates vasoconstriction, inflammation, and the proliferation of endothelial cells by interactions with NO [58]. *ET-1* expression is induced by TNF-α, tumor growth factor (TGF)-β, IL-1, ox-LDL, angiotensin II, and hypoxia, and regulated by several transcription factors, including activator protein 1, hypoxia-inducible factor-1, vascular endothelial zinc finger 1, and GATA-binding protein 2 [59]. ET-1 is formed by ET-converting enzymes and expressed in several cells, including endothelial cells, VSMCs, and macrophages. Two ET-receptor subtypes (ET_A_ and ET_B_) have been previously reported [60,61,62], with endothelial cells harboring ET_B_ receptors, and SMCs and macrophages expressing both receptors on the cell surface. ET-1 binds to ET_A_ receptors on VSMCs to induce vasoconstriction, and ET_B_ receptors on endothelial cells for vasodilatation by release of NO [63]. ET-1 expression can be both inhibited and stimulated by eNOS. In atherosclerotic arteries, ox-LDL stimulates the release and enhanced tissue levels of ET-1 in endothelial cells, VSMCs, and inflammatory cells [64,65]. High concentrations of ET-1 induce the expression of endothelial cell adhesion molecules and promote monocyte migration and activation regulated by MCP-1. Additionally, ET-1 stimulates VSMC proliferation, cytokine, and superoxide production in macrophages. After foam cell formation, local inflammation and ROS increase to facilitate lesion development [66]. In such circumstances, foam cells produce ET-1, which can act on macrophages by binding to ET_B_ receptors (Figure 2) [67,68].

## 4. Roles of Immune-Mediator Regulation

The early phase of atherosclerosis is considered an inflammatory response to ox-LDL [69]. In this phase, hypercholesterolemia conditions increase LDL infiltration and retention, leading to the activation of endothelial and inflammatory cells by the release of pro-inflammatory factors [70]. The expression of leukocyte adhesion molecules causes leukocyte infiltration and adhesion [71], after which leukocytes release chemoattractant stimuli, including chemokines. MCP-1 attracts leukocytes harboring chemokine receptor (CCR)-2, including monocytes and T and B cells [72,73]. Interferon (IFN)-γ-inducible protein 10 (or C-X-C motif chemokine 10 (CXCL10)), IFN-inducible T cell α-chemoattractant (or CXCL11), monokines induced by IFN-γ (Mig or CXCL9) selectively attract T and B lymphocytes bearing CXC receptor CXCR3 [74,75]. The fractalkine CX3CL1, which is a membrane-bound chemokine, also promotes CX3CR1^+^ monocytes [76]. Macrophages are major players in initial inflammation and innate immune responses [77].

### 4.1. Macrophages 

**Figure 3:**
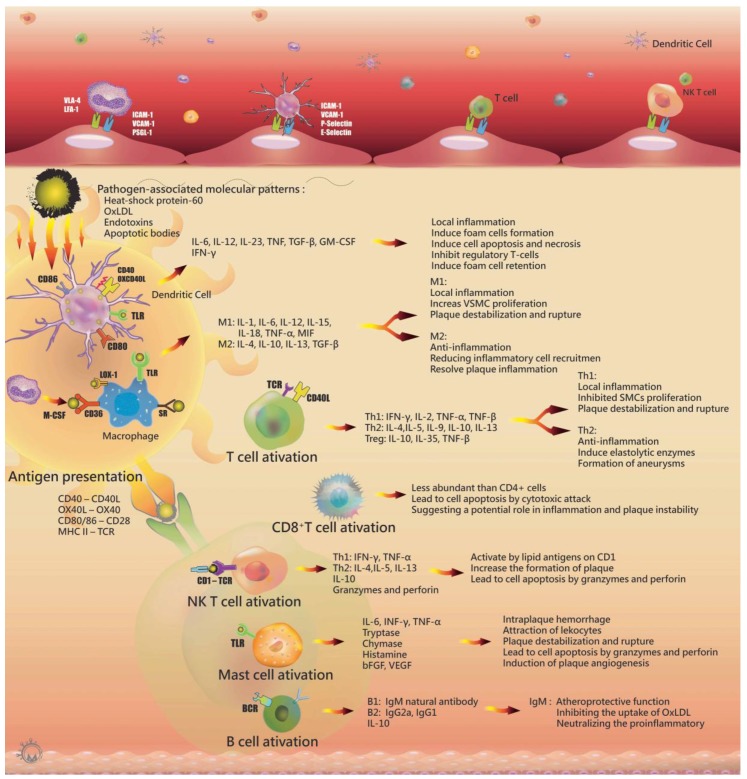
The role of inflammatory cells in atherosclerotic lesion.

Following exposure to chemoattractants and chemokines, monocytes become tethered via interactions between monocyte P-selectin glycoprotein 1 with endothelial P-selectins [78]. For adhesion and diapedesis, monocytes express the integrin very late antigen-4 and lymphocyte function-associated antigen-1 to bind to endothelial cell ligands, including VCAM-1 and ICAM-1 [79]. These monocytes differentiate into macrophages via monocyte colony stimulating factor (M-CSF) mediators. Macrophages use pattern-recognition receptors (PRRs), including SRs and TLRs, such as the transmembrane proteins SR-A (CD204), CD36, macrophage receptor with collagenous structure, and LOX-1 (OLR-1), to mediate the internalization of endotoxins, apoptotic bodies, and LDL particles [80,81]. After activation, monocytes differentiate to two main phenotypes of macrophages: M1 and M2 macrophages. Both inflammatory M1 and regulatory M2 macrophages are found in atherosclerotic lesions. M1 macrophages contribute to inflammation by secreting pro-inflammatory cytokines after intake of modified LDL and presenting antigen to T cells via PRRs, resulting in the release of pro-inflammatory cytokines, including IL-1, IL-6, IL-12, IL-15, IL-18, MIF, and TNF-α, to activate T cells. M2 macrophages have anti-inflammatory functions to resolve plaque inflammation by efferocytosis and releasing Th2 cytokines, such as IL-4, IL-10, and IL-13 [82] (Figure 3). 

In atherosclerotic lesion, the TGF-β from macrophages plays a role in vascular biology by affecting cell proliferation, differentiation, migration, adhesion, apoptosis, and extracellular matrix production [83]. TGF-β regulates both vasodilation and vasoconstriction via the TGF-β/ALK5/Smad3 pathway, inducing the expression of ET-1 on endothelial cells, and decreasing endothelial cell migration and proliferation [84]. However, TGF-β has a dual role in atherosclerosis [85]. It had a pro-atherosclerotic function by increasing VSMC proliferation [86]; while the anti-atherosclerotic processes from TGF-β involve reducing inflammatory cell recruitment, platelet adhesion, and macrophage activation [87]. Instead of either totally pro- or antiatherogenic function, TGF-β is demonstrated as having bifunctional effects on atherosclerosis [83,84,86,87].

TLRs also bind molecules and initiate a signaling cascade promoting macrophage activation to produce inflammatory cytokines, proteases, and cytotoxic oxygen- and nitrogen-radical molecules. Similar activities occur in dendritic cells (DCs), mast cells, and endothelial cells, which also harbor TLRs [88]. Vascular endothelial growth factor is also released from macrophages and promotes angiogenesis [89]. Cholesterol accumulation occurs in macrophages following ox-LDL uptake by SRs, including CD36, SR-A1, and LOX-1. The cholesterol esters are hydrolyzed, and ox-LDL molecules are catabolyzed by macrophage endosomes and lysosomes. In the endoplasmic reticulum, acyl coenzyme A cholesterol acyltransferase-1 esterifies the free cholesterol and stores it in lipid droplets. In atherosclerosis, lipid homoeostasis in macrophages is disrupted, causing the formation of foam cells. The apoptosis of foam cells is induced by prolonged endoplasmic reticulum stress due to their ineffective clearance by macrophages. This results in secondary cell necrosis, which subsequently forms a necrotic core. Macrophages trigger apoptosis in SMCs by activating the FAS apoptotic pathway and secreting proapoptotic TNF-α and NO. Macrophages also decrease collagen synthesis in SMCs and degrade various types of extracellular matrix via macrophage-derived MMPs, especially MMP-2 and -9. SMC death and a weakened extracellular matrix causes the rupture of atherosclerotic lesions, and the exposure of the thrombogenic material causes platelet adhesion and aggregation.

### 4.2. Dendritic Cells

Several studies support DCs as key modulators of immune responses and critical mediators of antigen presentation in atherosclerosis. DCs are a family of antigen-presenting cells that express high levels of major histocompatibility complex class II (MHC II) molecules and link innate and adaptive immune responses by presenting endogenous and exogenous antigens to T cells [90]. The life cycle of DCs involves three stages. In bone marrow, common dendritic precursor cells differentiate to classical DCs (cDCs) via FMS-like tyrosine kinase 3 (FLT3) and FLT3 ligand (FLT3L) signaling. cDCs differentiate into either lymphoid CD8^+^ or nonlymphoid CD103^+^ cDCs via activity associated with basic leucine zipper transcription factor ATF-like 3, interferon regulatory factor 8, and the inhibitor of DNA protein 2 [91,92,93,94,95,96]. In lymphoid tissue, CD8^+^/CD103^+^ cDCs are critical to the development of CD8^+^ DCs by cross-presentation of antigens to CD8^+^ T cells. CD4^+^/CD103^+^ cDCs also present antigen to CD4^+^ T cells. In nonlymphoid tissue, CD11b^+^ DCs are functionally similar to lymphoid CD4^+^/CD103^+^ cDCs, based on the expression of common macrophage markers, including signal-regulatory protein-α and F4/80. 

In atherosclerotic lesions, DCs are tethered to the activated endothelium with the help of adhesion molecules, including P-selectin, E-selectin, and VCAM-1 [97]. After the uptake of lipids and other antigens, DCs mature and present antigens in the context of co-stimulatory molecules, such as CD40, OX40L, CD80, and CD86 [98]. In lymphoid organs, DCs present the antigen to T cells and natural killer (NK) T cells in the context of MHC I, MHC II, or CD1d. [99] TLR interactions in DCs enhance antigen presentation and stimulate the production of a variety of inflammatory cytokines, including IL-6, TNF, IL-12, IL-23, and granulocyte M-CSF (Figure 3) [100]. Foam cells are phagocytized by macrophages or immature DCs [101], and after lipid uptake and efferocytosis, this leads to DC foam-cell formation. The inflammatory cells emigrate from the vessel wall to clear apoptotic cell debris and foam cells. Mature CD11b^+^ DCs silence regulatory T cells activated by ox-LDL, pathogen-associated molecular pattern, and/or damage-associated molecular pattern-related stress. DCs and T cell-derived IFNs induce inflammatory macrophages, DCs, and effector CD4^+^ T cell sensitization. Additionally, ox-LDL downregulates CCR7 expression and decreases the emigration rate of DCs. CCL17-producing DCs also inhibit regulatory T cells. This cycle increases foam cell retention, apoptosis, and necrosis [102,103].

### 4.3. T Cells

T cells are recruited to the atherosclerotic lesion in a similar mechanism as that of monocytes via several of the same adhesion molecules and chemokines. Most of these T cells are TCRαβ^+^ CD4^+^ cells found in human atherosclerotic plaques as compared with CD8^+^ or TCRγδ^+^ T cells [104,105,106,107]. T cells are activated by antigens presented from macrophages and DCs via two signals. First, ligations of the antigen receptor on the surface of antigen-presenting cells (APCs) initiate a response to pathogen-associated molecular patterns (PAMPs), which are molecules associated with pathogens. The MHC molecules interact with PAMPs, such as ox-LDL, microbial antigens, and heat shock proteins (HSP 60), which contribute to protect cells from stress damage expressed by stressed endothelial cells [108]. Second, the ligation of the co-stimulatory molecule CD28 to T cells allows interactions with CD80 or CD86 on APCs [109]. Additionally, T cells are also activated by interactions between CD40-ligand (CD40L or CD154) and CD40 on APCs, such as macrophages, B cells, DCs, endothelial cells, and SMCs. Early T lymphocyte activation protein-1 is an important stimulator of T helper cell 1 (Th1) differentiation, and is expressed by macrophages, endothelial cells, and SMCs in atherosclerotic lesions. Th1 also promotes IL-12 expression, which is also a stimulator of Th1 differentiation [110,111,112,113]. Th1 cytokines, including IFN-γ, IL-2, and TNF-α and -β, activate macrophages, endothelial cells, and SMCs and cause local inflammation. IFN-γ is a proatherogenic cytokine and growth inhibitor of endothelial cells and SMCs that also induces the production of inflammatory lipid mediators through their expression of secretory phospholipase A_2_ [114]. After arterial injury, SMCs are inhibited by IFN-γ secreted from T cells, which causes atherosclerotic plaque destabilization and rupture [115,116,117]. Additionally, IFN-γ augments TNF and IL-1 synthesis [118], which are powerful inflammatory inducers and indirectly inhibit the proliferation of SMCs and endothelial cells. Th1 cells activate signaling cascades related to pro-inflammatory cytokine expression, and macrophages are stimulated to secrete IL-1. These cascades involve the expression of TNF-α and IL-1 through -6 [119,120,121]. T cell cytokine expression causes the production of IL-6, which stimulates large amounts of acute-phase reactants, such as C-reactive protein, serum amyloid A, and fibrinogen, and results in local inflammation shifting to systemic reactions. The level of Th2-specific cytokine activity is lower than that from Th1 in atherosclerotic lesions, possibly due to local IL-12 secretion and IL-10 regulation [113,122]. Cross-regulation of Th1 and Th2 occurs through IL-10 inhibition of the Th1 pathway, with IL-12 as an inhibitor of the Th2 pathway. Although Th2-related cytokines are involved in anti-inflammatory activity, they also induce elastolytic enzymes to promote the aneurysm formation (Figure 3) [123,124]. 

### 4.4. Other Cells in Atherosclerosis

CD8^+^ T cells are a subpopulation of activated T cells that exhibit cytotoxic activity. In atherosclerotic lesions, after recognition of a foreign antigen from APCs, CD8^+^ T cells promote cell apoptosis via cytotoxic attack [125]. NK T cells are a minor T cell subpopulation that are activated by lipid antigens and recognized by CD1 molecules on APC surfaces. Recent studies support NK T cells promoting increases in the formation of atherosclerotic plaques [126,127,128,129]. After activation by glycolipid recognition by CD1, NK T cells product Th1 cytokines, such as IFN-γ and TNF-α, and release granzymes and perforin to promote apoptosis (Figure 3).

Mast cells and B cells are less frequently found in atherosclerotic lesions. Mast cells are classically involved in allergic and host defense responses. After activation, mast cells release pro-inflammatory cytokines and proteolytic enzymes, which contribute to atherogenesis. Mast cell interactions with APCs promote the production of TNF-α, INF-γ, and IL-6 [130,131]. Large concentrations of tryptase, chymase, the 5-lipoxygenase product leukotriene B4, and granulocyte M-CSF are released from mast cells in atherosclerotic lesions, leading to lesion destabilization and increased Th1 and Th2 responses. Additionally, activated mast cells correlate with higher levels of apoptosis, vascular leakage, and intraplaque hemorrhage and rupture [132,133].

Recent studies investigated the role of B cells in atherosclerosis. B cells are activated by foreign antigens via the B cell receptor, and B1 lymphocytes produce natural antibodies specific for ox-LDL, including Immunoglobulin M (IgM) antibodies, which might exhibit atheroprotective functions. The B2 subset of B cells, which is independent of Th1 cells, releases IgG2a and IgG1 [134,135,136]. Additionally, B cells also produce IL-10, which do not specifically affect Th1 or Th2 responses. Although some antibody reactions to atherosclerosis have been investigated in mice, B cell biology in human forms of the disease remains uncertain [123,137].

## 5. Plaque Rupture and Platelet Activation

Apoptotic cells in atherosclerotic lesions are ineffectively cleared by defective efferocytosis in macrophages, leading to secondary necrosis and the formation of the necrotic core [138]. The release of pro-inflammatory stimuli—including proteases, cytokines, and prothrombotic factors—from inflammatory cells increases collagen degradation or decreases collagen synthesis by SMCs and promotes the destabilization of atherosclerotic lesions [139]. Macrophages also activate the *FAS* apoptotic pathway and release TNF-α and NO to induce SMCs apoptosis. Macrophage-derived MMPs from macrophages and endothelial cells can weaken the fibrous cap [140], the rupture of which causes the exposure of thrombogenic material, which contributes to platelet aggregation and activation [141]. 

After disruption of the endothelium, platelets are recruited and tethered to activated endothelium via the interaction of platelet glycoprotein (GP) Ib-V-IX and von Willebrand factor (vWF) [142]. The platelets attach to the subendothelial matrix via GPVI, which acts as a collagen receptor and forms a complex with the receptor Fcγ. The VWF forms a complex with the GPIb-IX complex, which is associated via the platelet-adhesion mechanism [143]. After adhesion, platelets upregulate formation with the GPIIb-IIIa complex of stable platelet aggregates by binding multiple ligands, including fibrinogen, vWF, fibronectin, and vitronectin [144,145,146,147]. After stable adhesion, the platelets are activated and induce intracellular calcium flux to release α granules and dense granules, including 5-hydroxytryptamine, adenosine diphosphate, and adenosine triphosphate, for amplification of the platelet response. The α-granules include thromboxane A2, leukotriene B4 (LTB4), and a variety of pro-inflammatory mediators [148], which affect SMCs, endothelial cells, and promote cross-talk between monocytes and macrophages [149,150]. The release of IL-1β and expression of CD-40L induce endothelial cells to produce IL-6, IL-8, MCP-1, and ROS, as well as the upregulation of leukocyte-adhesion molecules. The cytokine cascade and LTB4 contribute to the release of chemoattractants and the promotion of monocyte differentiation and neutrophil activation, as well as the monocyte release of chemokines, enhanced cyclooxygenase expression, and overproduction of prostaglandin E_2_ and I_2_. After platelet stable adhesion, the thrombus may encroach into the vessel lumen and cause the ischemia or infarction. 

Another less common substrate for coronary thrombosis, plaque erosion, consists of a fibrous cap with lower cholesterol and a small or absent lipid core [151]. It is characterized by abundant surface smooth muscle cells and proteoglycans [152]. The mechanisms of plaque erosion are unclear. Current studies promote a hypothesis that the expression of TLR2 on endothelial cells may interact with Gram-positive toxins and hyaluronan, triggering endothelial dysfunction via ROS and cell apoptosis signaling. The endothelial damage may induce local inflammation and recruit neutrophils to attack endothelial cells [153]. Neutrophils are activated and release proteases to damage endothelial cells. After endothelial cell detachment, the exposure of the subendothelial matrix leads to thrombus formation [154]. Although plaque rupture and erosion may be caused via different mechanisms, both events may lead to infarction and ischemia due to the stenosis of vessel lumen.

Current drugs for controlling atherosclerosis majorly target the lipid metabolism and platelet activation pathway, such as station and aspirin. In particular, the statins regulate LDL metabolism and dramatically decrease the mortality and mobility of acute coronary syndrome. Nevertheless, mortality and mobility rates for acute coronary syndrome and stroke remain high in Westernized societies. Current studies support the hypothesis that inflammatory effector mechanisms play a critical role in atherosclerosis by the release of cytokines and inflammatory cell infiltration, leading to plaque rupture, erosion, thrombosis, acute coronary syndrome, and stroke. The anti-inflammatory drugs are new weapons to slow down the progression and development of cardiovascular disease. Although the detailed mechanisms are unclear, anti-inflammatory properties represent a promising new target against atherosclerosis.

## 6. Conclusions

In this review, we presented research associated with the roles of inflammatory cells and cytokines in atherosclerotic plaque initiation and progression. Numerous principles presented here are worth reiterating. First, hyperlipidemic status causes ox-LDL accumulation as the first step in atherosclerosis progression. Second, endothelial dysfunction impairs the regulation of vascular tone by increasing ET-1 levels and decreasing NO levels. Finally, macrophages play an important role in the inflammatory response, and following their activation, are involved with other immune cells in the advanced atherosclerotic lesion. The complicated mechanisms associated with inflammatory responses related to atherosclerosis remain largely unclear. This review provides an overview of recent studies of inflammatory responses related to atherosclerosis and offers a strong foundation for therapeutic interventions.

## Abbreviations

AGEs	Advanced glycation end products
ALK5	Transforming growth factor, beta receptor I
APCs	Antigen-presenting cells
Apo	Apolipoprotein
BCR	B cell receptor
bFGF	Basic fibroblast growth factor
BH4	Tetrahydrobiopterin
CD36	Cluster of differentiation 36
DCs	Dendritic cells
ECEs	ET converting enzymes
ET-1	Endothelin-1
eNOS	Endothelial isoform of NOS
GTP	Guanosine 5′-triphosphate
GTPCH I	Guanosine 5′-triphosphate cyclohydrolase I
HSP	Heat shock proteins
ICAM-1	Intercellular adhesion molecule-1
JNK	c-Jun N-terminal kinase
KLF2	Kruppel-like factor 2
LDL	Low-density lipoprotein
LFA-1	Lymphocyte function-associated antigen 1
LOX-1	Lectin-like oxidized LDL receptor-1
LPL	Lipoprotein lipase
LSS	Laminar shear stress
Ox-LDL	Oxidized LDL
MAPK	Mitogen-activated protein kinase
MCP-1	Monocyte chemoattractant protein-1
M-CSF	Macrophage colony stimulating factors
MHC-II	Major histocompatibility complex class II
MMPs	Matrix metalloproteinases
NADPH	Nicotinamide adenine dinucleotide phosphate
NK T cells	Natural killer T cells
NO	Nitric oxide
NOS	NO synthase
PDGF	Platelet-derived growth factor
PGE_2_	Prostaglandin E_2_
PGI_2_	Prostaglandin I_2_
PRRs	Pattern recognition receptors
PSGL-1	P-selectin glycoprotein 1
ROS	Reactive oxygen species
SMAD3	Mothers against decapentaplegic homolog 3
SMC	Smooth muscle cell
SR	Scavenger receptor
Th1	Type 1 T helper response
TG	Triacylglycerol
TLR	Toll-like receptor
TxA_2_	Thromboxane A_2_
VCAM-1	Vascular-cell adhesion molecule 1
VEGF	Vascular endothelial growth factor
VLA-4	Very late antigen-4
VLDL	Very low-density lipoproteins
VWF	Von Willebrand factor
